# Adapting a Counseling-Plus-mHealth Intervention for the Virtual Environment to Reduce Sexual and Reproductive Health Risk Among Young Women with Depression

**DOI:** 10.1007/s11121-023-01506-4

**Published:** 2023-03-07

**Authors:** Maddie O’Connell, Brittany Gluskin, Sarah Parker, Pamela J. Burke, Emily Pluhar, Carly E. Guss, Lydia A. Shrier

**Affiliations:** 1 Division of Adolescent/Young Adult Medicine, Boston Children’s Hospital, Boston, MA USA; 2 Bouvé College of Health Sciences, School of Nursing, Northeastern University, Boston, MA USA; 3 Department of Pediatrics, Harvard Medical School, Boston, MA USA; 4 Division of Sports Medicine, Department of Orthopedics, Boston Children’s Hospital, Boston, MA USA

**Keywords:** Adolescent, Young adult, Women, Telehealth, Sexual health, Sexual risk, Depression, Mental health, mHealth, Intervention

## Abstract

MARSSI (Momentary Affect Regulation – Safer Sex Intervention) is a counseling-plus-mobile health (mhealth) intervention to reduce sexual and reproductive health (SRH) risks for women with depression and high-risk sexual behavior. Due to the COVID-19 pandemic limiting in-person care, we sought to develop the counseling and mhealth app onboarding for virtual implementation. A team with SRH, adolescent medicine, motivational interviewing, cognitive behavioral therapy, and technology expertise adapted the counseling through an iterative consensus process. We identified essential aspects of the counseling, specified the content so the counseling could be delivered in person or virtually with fidelity, and considered best practices for telehealth for the focus population. Virtual counseling retained key elements from in-person counseling while including enhancements with engaging visual and audio–video aids. Instructions and programming were developed to support virtual counseling delivery and onboarding for the mhealth app component of MARSSI. After testing the virtual format in mock sessions, we implemented a small-scale feasibility study in an adolescent medicine clinic with women with depressive symptoms and high-risk sexual behavior age 18–24 years (*N* = 9). Participants experienced minimal technical difficulties and expressed satisfaction with the virtual format, and all were able to complete app onboarding successfully. Expanding delivery options for SRH interventions to include virtual can improve access, particularly for populations with psychological and environmental barriers to care.

## Introduction

Adolescent and young adult (AYA) women with depression have pronounced sexual and reproductive health (SRH) risk compared with their non-depressed counterparts (Hensel et al., 2016). AYA women with depression are more likely to engage in risk behaviors like condom non-use, sex with multiple partners, and sex under the influence of substances (Foley et al., 2019; Jackson et al., 2015; Shrier et al., 2009). They are also more likely to report no or inconsistent contraceptive use and less likely to report using long-acting reversible contraception (LARC) (Hall et al., 2013, 2014). As a result, AYA women with depression have a disproportionately higher risk for adverse SRH outcomes, including unintended pregnancy, unintended first birth, and sexually transmitted infections (STIs) (Hall et al., 2014; James-Hawkins et al., 2014; Lee et al., 2009).

In response to the lack of interventions that address both depression and SRH, a multidisciplinary team of adolescent health professionals developed the Momentary Affect Regulation – Safer Sex Intervention (MARSSI). MARSSI is a counseling-plus-mobile health (mhealth) intervention to reduce unintended pregnancy and STIs among AYA women with depression (Shrier et al., 2020). Acknowledging that low motivation is a core symptom of depression, MARSSI employs a Motivational Interviewing (MI)–based approach to enhance self-efficacy for changing SRH behavior (Treadway et al., 2012). To address negative affect in relation to SRH behavior, MARSSI incorporates well-established cognitive behavioral therapy (CBT) techniques, including cognitive restructuring (identifying and challenging unhelpful thoughts) (Klein et al., 2007). The intervention begins with an in-person “main” counseling session (~ 1 h) to elicit motivation for SRH behavior change and develop a plan for meeting behavior change goals, provide information on contraception and condoms, develop sexual communication skills, and teach cognitive restructuring. At the session, participants prepare to use the mhealth app by downloading the app onto their personal smartphone and indicating their preferences for voice and style of the app messages (“app onboarding”). Participants then complete 4 weeks of mhealth app reporting and messaging (4 times per day: 1 momentary report prompted at random within morning, afternoon, and evening time periods and 1 diary report at a scheduled time). Upon report of poor affect, low contraceptive or condom self-efficacy, pregnancy desire, or desire for sex to regulate affect, participants receive tailored messages in their preferred voice and style (Shrier et al., 2020). The intervention culminates in a “booster” counseling session (~ 30 min) to check in about goal progress and to teach a new skill. In a pilot study, conducted in 2017–2018, MARSSI demonstrated feasibility and acceptability with participants (Shrier et al., 2019, 2020).

The Coronavirus Disease 2019 (COVID-19) pandemic presented challenges for implementing MARSSI, including counseling and app onboarding, and more broadly limited access to in-person SRH care (Lindberg et al., 2020a, b; Mmeje et al., 2020). Pandemic-related social changes (e.g., isolation, school or workplace closure, illness or death of loved ones, reduced privacy at home, extended time at home) and economic changes (e.g., unemployment, economic insecurity) have been associated with an increase in depressive feelings among young women (Magson et al., 2021; Stavridou et al., 2020; Vindegaard & Benros, 2020; Wang et al., 2020). At-risk depressed AYA women may have been particularly impacted by the pandemic effects on mental health and on SRH care (Gluskin et al., 2022; Mmeje et al., 2020; Stavridou et al., 2020). The pandemic therefore produced greater need for interventions like MARSSI and, at the same time, created difficulties around its delivery.

In response to limitations on in-person healthcare, SRH providers have adopted telehealth not only as a necessity (Wilkinson et al., 2020), but also as a mechanism for continued care and increased access (Barney et al., 2020). SRH telehealth can reach AYA women who may not otherwise seek in-person care due to geographical barriers or stigma (Hubach et al., 2022; Murewanhema, 2020). For SRH services not requiring a physical exam (e.g., consultations, contraception prescriptions), telehealth shows promise as a tool for efficient and convenient healthcare delivery (DeNicola et al., 2020; Shaikh et al., 2021; Stifani et al., 2021; Williams et al., 2018), despite potential barriers such as privacy risks, technical challenges, or lack of patient access to (quality) internet or technology (Bacchus et al., 2019; Faccio et al., 2021; Kaufman et al., 2016). AYA may be particularly accepting of telehealth owing to their familiarity with technology (Anderson & Jiang, 2018). For delivering telehealth services to AYA with depression, additional telehealth considerations include provider rapport and safety procedures (Connolly et al., 2020; Cowan et al., 2019; Montoya et al., 2022; Reay et al., 2020). There is limited research on the feasibility of telehealth for AYA women with depression and SRH risk.

With careful consideration of the advantages and challenges of SRH telehealth, especially for AYA with depressive symptoms, MARSSI may be particularly well-suited for adaptation to the virtual environment. Major components of MARSSI already rely on virtual delivery: participants engage with an mhealth app, and a videoconference option for the booster counseling session had been offered in the prior iteration of MARSSI (Shrier et al., 2020). In this study, we adapted the in-person MARSSI counseling sessions and mhealth app onboarding for fully virtual implementation, and we developed a safety plan for the virtual setting. We subsequently sought to determine the feasibility of the virtual adaptation of MARSSI in a small-scale trial with patients of an urban adolescent medicine clinic.

## Methods

### Virtual Adaptation

Our team with SRH, adolescent medicine, MI, CBT, and technology expertise developed virtual intervention materials and procedures from July to December 2020 via an iterative consensus process; consensus methods emphasize group agreement for decisions on topics that lack empirical evidence (Waggoner et al., 2016). In September and October 2020, the research team solicited feedback from 3 high school age youth advisors (2 female, 1 male) affiliated with the researchers’ home institution. Research staff met with youth advisors in two virtual feedback sessions (45 and 30 min, respectively), in which the advisors offered input on implementation procedures (e.g., venues for virtual recruitment, access to technology and virtual materials) and intervention content (e.g., design and presentation of virtual materials, new materials not previously tested). A trained research team member facilitated each session while another team member documented minutes and a third team member observed. In keeping with the iterative consensus process, the research team then discussed the youth advisors’ key points and suggestions, considered the implications of development decisions vis-à-vis research implementation, and arrived at final decisions by team agreement.

The development process consisted of 5 stages:

1. We identified essential aspects of the in-person counseling by considering the MARSSI logic model, the MI and CBT approaches used in MARSSI, and the results of a pilot test of the in-person form of the intervention (Shrier et al., 2020).
2. We identified and customized technological tools (i.e., programs and applications) that would facilitate virtual intervention delivery. These tools were selected on the basis of their ability to maintain the essential counseling aspects, enable privacy and security safeguards (as established by the research team), and offer ease of use (as supported by feedback from youth advisors and a trained MARSSI counselor).
3. We specified intervention content and instructions so that counseling can be delivered in person or virtually with fidelity. We assessed intervention content, activity-by-activity, and overall, for its appropriateness in the virtual environment, making enhancements as needed. As individual counseling activities were adapted to a virtual format, the research team iteratively made improvements and sought feedback from youth advisors.
4. We considered best practices for telehealth, especially with regard to the focus population, who require mental health safety considerations. We solicited from youth advisors what youth might need to participate in the telehealth environment. We consulted with clinic leadership in mental health (a social worker) and medicine (a physician) about existing telehealth safety procedures; team members with clinical expertise developed study-specific virtual safety procedures to align with existing clinic practices.
5. We performed and video-recorded mock counseling sessions between a counselor who was trained to deliver the intervention in person and 5 volunteers (including non-project staff; ages ranging from late teens to mid-20 s) acting out standardized participant cases, with a research team member observing. After the mock sessions, the volunteers discussed their experience as mock participants, then the research team reviewed the recordings for virtual delivery flow and function. We then made revisions to intervention procedures.

### Feasibility Trial

#### Recruitment

Patients of an adolescent medicine clinic at a large urban children’s hospital were recruited between March 2021 and April 2022. Patients were invited to self-screen via a survey link and QR code displayed on flyers hung throughout the clinic. Patients who met age and sex eligibility criteria through electronic medical record screen were sent information about the study directly via patient portal or referred to the electronic self-screening survey by their clinician during health visits.

#### Participants

Eligibility criteria were assigned female sex at birth and able to become pregnant; currently sexually active, defined as penile-vaginal sex ≥ 1 time per week, on average (Shrier et al., 2012)^1^; at least 1 increased pregnancy/STI risk behavior in the past 3 months (low effectiveness/inconsistent/no birth control use, inconsistent/no condom use, two or more sexual partners, sex within 2 h of alcohol or other drug use, receipt of STI treatment); and aged 18–24 years. Although MARSSI is intended for AYA women 15–24 years old, we chose to recruit only adults while we developed and tested our safety plan. Eligible patients also met criteria for clinically significant depressive symptoms, defined as a Patient Health Questionnaire-8 (PHQ-8) score ≥ 8 (Kroenke et al., 2009), an acceptable diagnostic cutoff score (Manea et al., 2012) that enhances the sensitivity of screening for subclinical depression as well as depressive disorders (Zuithoff et al., 2010).

Based on our formative work, patients were excluded if they were currently pregnant or trying to become pregnant, had given birth in the past 6 months, were married or engaged to be married, could not communicate fluently in English, and/or had previously participated in MARSSI research (Shrier et al., 2020). All respondents, eligible or not, earned a $5 Starbucks e-gift card for completing the screening survey. Eligible patients were invited to enroll in the study. Of 145 patients who completed the electronic self-screening survey, 19 were eligible and 9 enrolled (47.4% of eligible patients). The primary reason for ineligibility was not being currently sexually active (94 patients). Of the 10 eligible patients who did not enroll, 2 were not interested, 2 did not respond to contact attempts, and 6 were contacted but lost to follow-up during scheduling.

#### Procedures

Study visits were conducted using a secure videoconferencing platform available to participants at no cost (Zoom™). All assessments were administered electronically through links placed in the videoconferencing chat or text messaged to participants. Prior to starting Study Visit 1, the research coordinator obtained the patient’s informed consent to participate via electronic consent (e-consent) form. The participant then completed a baseline survey on demographic characteristics and their sexual behavior and emotional health, met with the study counselor for the main counseling session, and completed a post-session survey on the counseling experience. At the end of Study Visit 1, the research coordinator provided the participant a link to download the mhealth app and take a survey soliciting their message preferences, as previously described (Shrier et al., 2020). The participant was asked to respond to app surveys and receive messages for the next four weeks.

After the 4 weeks of app use, the participant returned for a virtual Study Visit 2 to complete a follow-up survey on their sexual behavior and emotional health, meet with the study counselor for the booster counseling session, and complete a post-session survey on the counseling experience. Two weeks after Study Visit 2 (6 weeks after enrollment), the participant completed a post-intervention survey on their depressive symptoms and self-efficacy and motivation related to changing their SRH risk behavior. All participants completed the main counseling session and 7 completed the booster counseling session (1 declined a booster for scheduling reasons, 1 was lost to follow-up). Participants were compensated with up to $75 in Amazon e-gift cards based on their completion of the study activities. This study was approved by the hospital Institutional Review Board.

### Feasibility Measures

In this paper, we report on the feasibility measures assessed on the post-session experience surveys and through observation. Measures focused on aspects of feasibility that might differ from original pilot testing as a result of the virtual adaptation.

#### Practicality

We measured participant completion rates of the main and booster counseling, and duration in minutes of each counseling sessions. On the survey following each counseling session, participants reported occurrence(s) of technical difficulty (1 multiple response item with choices such as “My video froze,” “My counselor’s video froze”). If any technical difficulties occurred, participants indicated to what extent the issues negatively impacted the session (1, Not at all, to 5, A great extent).

#### Acceptability

After each virtual counseling session, we asked participants to rate characteristics and perceived helpfulness of the counselor interaction, the individual session activities, and the session overall (21 items; 1, Strongly Disagree, to 5, Strongly Agree). We also asked participants to select all that apply from a list of adjectives to describe each session (e.g., “Boring,” “Informative,” “Fun”). After the booster session, participants rated the quality of the entire MARSSI program (1, Poor to 5, Excellent), and indicated whether they would recommend MARSSI to other people their age (Yes, No, Maybe).

#### Implementation

To determine app engagement following virtual onboarding, we calculated the percentage of days on which participants completed ≥ 1 app survey. Additionally, we monitored implementation of the study safety plan.

##### Feasibility Analysis

To characterize feasibility results, we used descriptive statistics appropriate to the small sample size. We generated counts and/or medians and interquartile ranges (IQR) to describe sample demographics, eligibility criteria, and report of technical difficulty. We computed means and standard deviations (SD) to illustrate the remaining practicality and acceptability outcomes.

## Results

We report outcomes from each development stage, including final products and virtual delivery processes, as well as informative youth feedback. We also present findings from the feasibility study.

### Virtual Adaptation

#### Stage 1: Identify essential aspects of the in-person counseling.

The research team identified five essential aspects of in-person counseling.

1. An MI-informed approach: MARSSI’s approach is conceptually informed by MI, a participant-centered counseling style (Miller & Rollnick, 2012), to enhance motivation to change SRH behavior. Participants of in-person MARSSI had identified the MI approach as “collaborative” and reported increased confidence to reduce their SRH risk behavior after counseling (Shrier et al., 2020). The research team, including an MI expert, identified the MI-informed approach as essential because it fosters a collaborative, non-judgmental environment in which the participant’s autonomy and self-determination are regarded as integral to making a change to their sexual behavior.
2. 1:1, face-to-face delivery: MARSSI utilizes a counseling-plus-mhealth approach because momentary interventions may be more effective when integrated with face-to-face counseling (Versluis et al., 2016). The research team identified 1:1, face-to-face delivery as essential because it builds counselor-participant rapport and establishes a trusting relationship for the shared discussion of sensitive topics, both being tenets of MI.
3. CBT skill-teaching: MARSSI addresses the affective and cognitive determinants of SRH by teaching participants cognitive restructuring, a CBT skill. Participants of the in-person counseling described this CBT-based skill-teaching activity as a “good idea,” and nearly all (94.1%) participants reported using the cognitive behavioral skill at least once in the 4 weeks following main counseling (Shrier et al., 2020). The research team, including a CBT expert, identified CBT skill-teaching as essential because it supports the participant’s ability to recognize and address the unhelpful thinking patterns that may characterize the relationship between their depressive symptoms and their sexual behavior.

Additionally, based on expert consensus and previous feedback from in-person participants that “the SRH content was appealing and useful” (Shrier et al., 2020), the research team identified the following educational approaches as essential:

4. Interactive activities, to engage the participant and build their skills and self-efficacy to make a change (e.g., roleplaying a scenario in which the participant and a partner discuss condom use).
5. Handouts, to provide the participant with evidence-based information (e.g., effective contraceptive methods) that can be referenced after the counseling.

#### Stage 2: Identify and customize technological tools that maintain essential counseling aspects and are easy to use.

Table 1 depicts MARSSI’s specific needs for the virtual environment—derived from MARSSI’s five essential counseling aspects—and the technological tools selected to achieve those needs. Counselor and youth feedback indicated that ease of use constituted (1) familiarity with the selected tool, (2) simplicity of the selected tool (e.g., for one youth advisor, “easy to navigate”), and (3) the ability for each of the selected tools to interface smoothly. We chose to minimize the number of tools to reduce the possibility for technical issues. This also allowed the counselor to focus on delivering the intervention holistically, as the technology is the means by which MARSSI is delivered, not its focus.

**Table 1:** Technological tools for delivering essential MARSSI counseling aspects and needs in the virtual environment

**Essential counseling aspect**			**Specific need for the virtual environment**	**Tool selected**	**Rationale for tool selection**
1:1, face-to-face delivery MI-informed approach Interactive activities	CBT skill-teaching		Audio–video delivery format	Videoconference via Zoom™	• Secure platform approved by the study team’s home institution • Password-protected meetings, participant waiting room • Maintains generally reliable connection, picture clarity • Allows the counselor to share their computer screen, audio • Offers engaging annotation functions (e.g., text boxes, stamps, free drawing) during screen-share • Recording capabilities
		Handouts	Ability to share and present materials during the session	Presentation of handouts using Google Slides™	• Free and familiar presentation tool • Offers attractive slide design options • Restricted user access • Advanced slide functions (e.g., skip logic between slides) • Web-based, synchronized version control, i.e., all MARSSI team members always have access to the same, current slide set
			Availability of materials after the session	Study website serving as digital repository, hosted by Wix^®^	• Relatively affordable compared to other web design platforms • User-friendly • Allows for password-protected web pages that are hidden from search engine optimization (e.g., Google). This minimizes the risk of contaminating control participants with intervention materials and revealing counselor materials to participants

*MARSSI*, Momentary Affect Regulation + Safer Sex Intervention

Zoom™ is a trademark of Zoom Video Communications, Inc. Google Slides™ is a trademark of Google LLC. Wix^®^ from Wix.com, Inc.

#### Stage 3: Specify intervention content and instructions so that counseling could be delivered in person or virtually with fidelity.

Table 2 depicts the specific considerations and adaptations for individual activities within MARSSI counseling. For activities that previously involved paper handouts, we adapted the handouts to electronic form, building them into the counseling slide set via Google Slides™, screen-sharing the slide set from Zoom™, and posting downloadable PDF files to a study website via the Wix^®^ web design platform. The latter aligned with input from youth advisors, who emphasized that access to materials after a session was “important,” and that provision of materials through traditional mail—though it should remain an option—could be “too slow” and “too public” (i.e., risks revealing participation).

**Table 2:** Virtual adaptation of MARSSI counseling activities

**Activity name**	**Activity description**	**Key considerations from in-person counseling for virtual adaptation**	**Virtual adaptation of activity**
**Using Effective Contraception**	Increase knowledge of birth control options	Participant engages with informational handouts	Counselor screen-shares handouts and optionally clicks out to contraceptive resource website (www.bedsider.org).
**Choosing Not to Have Sex**	Enhance ability to say no to sex and explore other ways to feel good that do not involve sex (self-soothing)	Participant identifies effective method(s) for saying no, self-soothing from lists of examples	Counselor screen-shares handouts.
**Using Condoms Correctly and Consistently**	Build self-efficacy for condom use	Counselor demonstrates condom application and provides gift (condom keychain or condom compact)	Pre-recorded video of correct application presented alongside a checklist and with counselor narration; participant or counselor uses electronic stamp to check off each list item on shared screen. Counselor asks permission to mail gift to participant.
**Game Plan**	Plan steps for meeting behavior change goal	Participant and counselor both retain copy of plan for reference at booster session and general future reference	Counselor annotates participant’s responses in a text box overlaying handout on shared screen and uses electronic stamps (e.g., star, heart) to mark participant’s key ideas. Counselor saves screenshot and sends electronic copy to participant.
**Motivation Rulers**	Assess importance, readiness, and confidence for making behavior change	Participant sees and chooses number from 0 to 10 on each ruler	Counselor screen-shares each individual ruler and uses electronic stamps (e.g., star, heart) to mark chosen numbers.
**Having a Healthy Relationship(s)**	Assess and reflect on the components of healthy intimate relationships	Participant reviews list of things that apply to their current intimate relationship(s)	Counselor screen-shares handout; as participant verbalizes answers, counselor or participant uses electronic check mark stamp to mark list items that apply.
**Managing Unhelpful Thoughts**	Build CBT skills	Participant applies Catch It – Check It – Change It cognitive restructuring technique	Counselor annotates participant’s responses in a text box overlaying handout on shared screen.
**My Affirmations**	Practice affirmations	Participant repeats affirmation while looking at own mirrored reflection	Participant looks at own video tile.

*MARSSI*, Momentary Affect Regulation + Safer Sex Intervention; *CBT*, cognitive behavioral therapy

We examined our handouts for legibility and clarity when screen-shared and viewed electronically, adjusting details like font, layout, and color to enhance handout quality. We replicated interaction with paper documents using simple technical features of the videoconferencing platform. For example, when the participant is brainstorming a “game plan” for changing a sexual risk behavior, the counselor can use the annotation feature to type the participant’s responses in text boxes on the shared screen and use stamps (e.g., stars, check marks) to indicate the participant’s key ideas (Table 2). For some activities, the participant has the option of annotating the screen themselves, such as using check mark stamps to identify healthy characteristics of their intimate relationship(s) (Table 2).

In addition to maintaining fidelity to the content of the in-person intervention, we included audio and video to enhance the activities and counseling quality. For example, instead of a live condom demonstration on a penis model, based on feedback from youth advisors, we recorded a demonstration video to play alongside an electronic checklist. The counselor narrates the video while the participant checks off the steps observed in the video (Table 2). This format helps to maximize screen “real estate” and ensures that each participant can clearly see and actively engage with the condom demonstration activity. Youth advisor feedback also influenced our decision *not* to include a pre-recorded, externally developed video related to a CBT concept explored in the intervention: they “[did] not know how helpful it would be” and preferred the counselor to explain this concept in dialogue with the participant.

The development team’s CBT expert pre-recorded two brief audio relaxation exercises that were new to the intervention. The CBT-based exercises use a “grounding” technique to redirect attention from strong negative feelings to one’s physical state and surroundings in the present moment, which can decrease distress and increase calm. These optional exercises were developed to help participants to manage their emotional response to discussions about sexual health and depression. The audio exercises were perceived favorably by youth advisors, who offered the additional suggestion to play the audio exercise while screen-sharing a relaxing image. In response, the research team identified two relaxing GIFs (e.g., ripples in a pond) and built them into the slide set with the audio exercises.

#### Stage 4: Consider best practices for telehealth.

The research team, including the trained MARSSI counselor, agreed that developing clear virtual delivery guidance was essential. Anticipating varied levels of technical expertise among future counselors—and aiming for standardized virtual delivery procedures to maintain intervention fidelity—the development team created thorough technical instructions as part of a comprehensive counselor guide. The technical instructions contained both text and images to enhance learning. They intended to orient future counselors to virtual intervention delivery and tools (i.e., Zoom™, Google Slides™, Wix^®^ website; app onboarding) and equip counselors with troubleshooting tips in the event of a lapsed internet connection or other common technical issue.

The youth advisors similarly recommended that the study team provide clear technical instructions to participants. Although many youth had likely become experienced with videoconferencing from remote learning and work during the COVID-19 pandemic, the advisors anticipated that not all youth will have had experience with our chosen videoconference platform. The resulting participant technical instructions were brief, specific to Zoom™, and available in two versions, depending on how the participant logs on, by smartphone/tablet or by computer. The instructions shared pertinent technical tips through text and screenshots, including how to use special functions (e.g., chat, annotation), how to maximize privacy (e.g., using headphones), and how to minimize distraction (e.g., enabling “Do Not Disturb” device setting). Furthermore, the participant instructions were built directly into the counseling slide set—as opposed to a separate document—so that the counselor could easily share their screen with these tips at the beginning of the session.

Given MARSSI’s focus on depressed AYA women and our intended clinical setting for the feasibility trial, developing a virtual mental health safety plan was also identified as essential. The safety plan addressed (1) a score of 15 or higher on the PHQ-8 on the eligibility survey or post-intervention survey, indicating moderately severe or higher level of depressive symptoms (Manea et al., 2012) and consistent with clinic protocols for scores prompting outreach by a mental health clinician, and (2) spontaneous disclosure of a safety concern in the virtual counseling session (e.g., acute suicidality). For the survey-based safety flag, the safety plan instructed the research coordinator to report the concern to the participant’s primary care provider (PCP) within one business day by email with reply requested. Due to the short study window, the research coordinator only reported a participant’s first instance meeting the criterion. For safety concerns spontaneously disclosed during counseling, the plan instructed the counselor (a nurse practitioner) to use her clinical judgment to assess the situation. Depending on the severity and urgency of the safety risk disclosed, the safety plan outlined that the counselor should either (a) offer to pause the counseling and ask permission to continue, (b) stop the session and ask permission to reschedule, or (c) stop the counseling and have the on-call behavioral health clinician join the session, consistent with the site clinic’s usual practice for addressing immediate mental health concerns in telehealth visits. For non-emergent concerns, the counselor would also notify the participant’s PCP and recommend to participants that they follow up with their behavioral health clinician, if they had one.

#### Stage 5: Perform and record mock sessions between a trained counselor and volunteer actors to assess virtual delivery flow and function.

The study counselor completed nearly 15 h of mock testing with the 5 young adult volunteers. Post-mock session revisions included technical and design changes to handouts and activities in the slide set, aiming to improve aesthetics, flow, and legibility. Feedback also inspired changes to the counseling manual, such as incorporating up-to-date terminology, improving transitions between sections, and clarifying instructions. For example, in an early mock session, both the counselor and the volunteer expressed confusion around a CBT-based counseling section: the volunteer had trouble improvising responses to the counselor's scripted prompts, and the counselor subsequently struggled to navigate the activity. As a result, guided by the research team’s CBT expert, we made appreciable changes to the activity, which included scripting new prompts and instructions in the counseling manual; re-designing and animating the CBT handout, so that the counselor could guide the participant through the activity one step at a time rather than all at once; and adding a standardized example of a “participant” who completed the activity to prime participants for completing the activity themselves. Additionally, after technical issues arose, including unstable internet connection and screen freezing, we clarified the virtual delivery guidance to best troubleshoot these issues in the future.

### Feasibility Trial

Median baseline age was 21 years (IQR: 18–21 years); 4 participants self-identified as Black or African-American, 3 as White, 1 as Hispanic or Latinx, and 1 as White, Hispanic or Latinx. For the past 3 months, participants most frequently reported sex without a condom (*n* = 8) and sex within 2 h of substance use (*n* = 6), followed by use of less-effective contraception (*n* = 3), sex with multiple partners (*n* = 2), and STI treatment (*n* = 1). Median PHQ-8 score among participants was 13 (IQR: 12–16).

Median duration of main counseling was 68 min (IQR: 65–94 min); booster counseling, 38 min (IQR: 36–43 min). All sessions were completed in full. No participants elected to use the optional audio relaxation exercises during counseling. Participant-reported technical difficulty was uncommon during the virtual counseling (main *n* = 3; booster *n* = 1). Technical issues primarily comprised of frozen video, with 2 reporting that their own video froze, and 1 also reporting that their counselor’s video froze. One participant reported audio pausing or lagging. For all technical difficulties during counseling, participants reported minimal impact. All three participants with technical difficulties during the main session returned for the booster session.

Following the main counseling session, participants rated counselor interaction, perceived helpfulness of the session, and session activities a mean item score of 4.8 out of 5 (SD: 0.1) and rated the overall session 4.3 out of 5 (SD: 0.9) on average. Likewise, participants reported a mean item score of 4.9 (SD: 0.1) on booster counseling characteristics, scoring the overall session 4.8 (SD: 0.8) on average. Participants described the counseling sessions as “informative” (main *n* = 9; booster *n* = 6), “useful” (main *n* = 7; booster *n* = 5), and “fun” (main *n* = 7; booster *n* = 5); no participants characterized the counseling sessions as “boring,” “stupid,” or a “waste of time.” Two participants also rated the main counseling session “difficult,” and 1 participant rated the booster as such. All participants who completed the booster session indicated that they would recommend the program to a friend.

All 9 participants successfully onboarded to the app during Study Visit 1. Participants completed ≥ 1 app report on a median 82% of days (23 of 28 days) during the app period (IQR: 75–100%). Four participants completed ≥ 1 app survey every day during the app period (100% of days, 28 of 28 days) and 3 additional participants completed surveys on ≥ 75% of days (≥ 21 of 28 days); 1 participant did not complete any app surveys after Week 1.

The study safety plan was implemented only in respect to survey-based safety flags: 43 individuals who took the eligibility screening survey (30%) had a PHQ-8 score ≥ 15, resulting in notification to their PCP. Four participants who had not previously met this criterion at screening met it on the post-intervention survey. There were no serious safety concerns (e.g., suicidal ideation) disclosed during counseling sessions.

## Discussion

In this study, we successfully adapted MARSSI in-person counseling and app onboarding procedures to the virtual environment through an iterative consensus process. The virtual adaptation retains key characteristics of MARSSI counseling, including face-to-face discussion, verbal collaboration for developing a change plan, and sharing of educational materials. The technological platforms utilized by MARSSI underwent thorough, collaborative review and testing, and offer creative yet simple means for participant engagement, such as share-screen and screen annotation via teleconference, visually appealing virtual handouts in a pre-built slide set, and easy-to-navigate downloadable materials on the study website. The safety plan outlined procedures for linking participants to clinical care for high level depressive symptoms and suicide risk in a manner appropriate for the virtual setting. Mock testing generated useful feedback for finalizing the virtual intervention. As evidence of feasibility, participants rated the remotely delivered program very favorably and demonstrated near-daily app engagement after virtual app onboarding, similar to results achieved with in-person counseling and app onboarding in prior research (Shrier et al., 2020).

The virtual format is highly suited to the AYA members of MARSSI’s focus population, who are likely to have reliable access to technology and be technologically competent (Anderson & Jiang, 2018). Comfort with various aspects of videoconference healthcare, including speaking with a provider about sensitive health topics and having sufficient privacy to do so, has also been reported by young adult women both with and without depressive symptoms (Gluskin et al., 2022). MARSSI participants expressed high satisfaction with the virtual program, including counselor interaction, indicating good rapport. Although there were occasional technical issues, AYA familiarity with technology may have helped to minimize their impact. For participants needing orientation to our platforms (e.g., Zoom™), the virtual adaptation of MARSSI includes technical instructions.

In expanding options for program delivery to include virtual, we hope to reduce barriers to in-person SRH care for AYA with depressive symptoms, including access to youth-friendly providers and services, concerns regarding confidentiality and privacy, and challenges related to transportation. With virtual SRH care, patients may connect with health providers, services, or programs that are not otherwise available in their area, particularly in rural areas (Yoost et al., 2017). Patients have a degree of autonomy when choosing when and where to participate in a virtual visit, thus avoiding confidentiality concerns among younger adolescents, like presence of a parent/guardian in the exam room (Fuentes et al., 2018). Virtual visits have unique benefits that in-person visits lack, such as zero commute time, that support increased healthcare access (Brophy, 2017). Furthermore, the virtual format can eliminate the real or perceived stigma of visiting or being seen at an SRH clinic (Hubach et al., 2022). Expanding options for counseling delivery will improve access to SRH interventions, particularly for populations with psychological as well as environmental barriers to care (Cheng et al., 2021; Mmeje et al., 2020; Roth et al., 2019; Yoost et al., 2017).

MARSSI’s tailored focus on AYA women’s SRH and depression, when offered virtually, can also reduce barriers to care related to poor mental health. Compared with non-depressed youth, depressed youth have been likelier to perceive barriers to mental health care (Meredith et al., 2009). In addition to shared barriers with in-person SRH care, including geography, availability of services or providers, and stigma, depressed AYA may feel resistant or unmotivated to seek mental health care or lack knowledge around the variety of mental health care services available to them (Fleming et al., 2012; Radez et al., 2021). Virtual MARSSI is an opportunity to bring tailored care to AYA women, “meeting them where they are” to bridge gaps in care.

Our development process and feasibility test had limitations. Tools and platforms used for MARSSI (e.g., Wix^®^) may be cost-prohibitive for others to utilize in their own interventions, depending on the funding and resources available. Some technical issues are not preventable and may impact session quality, although our study suggests that those impacts may be minimal. The safety plan was specific to our recruitment site and the counselor’s training as a nurse practitioner; virtual interventions addressing sensitive topics that are delivered outside of a clinical context may require different plans that leverage the safety expectations of counselors, participants, and settings. In the feasibility trial, we experienced difficulties scheduling eligible patients; patients who were scheduled and enrolled may have been more likely than those who did not to report favorable feasibility ratings and to complete the sessions. Likewise, we experienced a high attrition rate; participants retained for the full study window may have been more satisfied with the program than those lost to follow-up. Because participants did not opt to use the audio relaxation exercises, we could not assess their feasibility or acceptability. We enrolled a small sample from one clinic within a large urban hospital, introducing the possibility of selection bias; a larger and more diverse sample is needed to better understand participant experience with MARSSI’s virtual form.

Strengths of this study include our multidisciplinary team and iterative consensus process, which allowed for thorough, continuous, and robust feedback and for applying knowledge from prior experiences developing and testing SRH interventions (Shrier et al., 2001, 2020), as well as aligned with recommendations from developers of other technology-based SRH interventions (Krishnamurti et al., 2022; Parekh et al., 2021). We received feedback from youth advisors, which informed the favorability, literacy, and attractiveness of materials. Mock testing allowed for improvements and enhancements before real participants were exposed to the virtual intervention. This comprehensive development process culminated in a feasibility test with individuals from the focus population. Further testing is needed to evaluate the sustainability and scalability of MARSSI’s virtual form. MARSSI is currently being implemented in a large-scale randomized control trial by an independent evaluator, which will also generate quantitative acceptability feedback and provide data on effectiveness.

## Conclusion

Our adaptation of MARSSI, feasible for the virtual format, demonstrates that in-person SRH counseling can be developed for remote delivery, retaining key elements while also enhancing the counseling with engaging visual, audio, and video aids. Strategies used to adapt and enhance MARSSI, if applied to other counseling-based interventions, can expand options for tailored SRH care, particularly for underserved and high-risk populations such as AYA women with depression.

## Data Availability

The data that support the findings of the feasibility study are available upon request from the corresponding author and approval from the study's Principal Investigator.

## Footnotes

1 In a previous study using this requirement to recruit a similar sample of depressed AYA, we found that participants had a median of 3.5 sex events per week, exceeding the lowest threshold of this criterion (Shrier et al., 2012). This criterion ensures that MARSSI is delivered to participants who are *currently* sexually active, for whom the intervention is designed.
